# Defining a malaria diagnostic pathway from innovation to adoption: Stakeholder perspectives on data and evidence gaps

**DOI:** 10.1371/journal.pgph.0002957

**Published:** 2024-05-16

**Authors:** Bryony Simmons, Elisa Sicuri, Jane Carter, Asrat Hailu, Francois Kiemde, Petra Mens, Davis Mumbengegwi, Bakri Nour, René Paulussen, Henk Schallig, Halidou Tinto, Norbert van Dijk, Lesong Conteh

**Affiliations:** 1 LSE Health, London School of Economics and Political Science, London, United Kingdom; 2 ISGlobal, Hospital Clínic Universitat de Barcelona, Barcelona, Spain; 3 Amref Health Africa Headquarters, Nairobi, Kenya; 4 Addis Ababa University, Addis Ababa, Ethiopia; 5 Clinical Research Unit of Nanoro, Institut de Recherche en Sciences de la Santé, Nanoro, Burkina Faso; 6 Amsterdam Institute for Immunology and Infectious Diseases, Infectious Diseases Programme, Amsterdam, The Netherlands; 7 Amsterdam University Medical Centre, Laboratory for Experimental Parasitology, Department of Medical Microbiology and Infection Prevention, Amsterdam, The Netherlands; 8 Centre for Research Services, University of Namibia, Windhoek, Namibia; 9 Blue Nile National Institute for Communicable Diseases, University of Gezira, Wad Medani, Sudan; 10 Mondial Diagnostics, Amsterdam, The Netherlands; 11 Department of Health Policy, London School of Economics and Political Science, London, United Kingdom; Menzies School of Health Research: Charles Darwin University, AUSTRALIA

## Abstract

Malaria, a major global health concern, requires effective diagnostic tools for patient care, disease control, and elimination. The pathway from concept to the adoption of diagnostic products is complex, involving multiple steps and stakeholders. To map this process, our study introduces a malaria-specific diagnostic pathway, synthesising existing frameworks with expert insights. Comprising six major stages and 31 related activities, the pathway retains the core stages from existing frameworks and integrates essential malaria diagnostic activities, such as WHO prequalification processes, global stakeholder involvement, and broader health systems considerations. To understand the scope and availability of evidence guiding the activities along this pathway, we conducted an online survey with 113 participants from various stages of the malaria diagnostic pathway. The survey assessed perceptions on four critical attributes of evidence: clear requirements, alignment with user needs, accuracy and reliability, and public and free availability. It also explored the types of evidence used and the challenges and potential solutions related to evidence generation and use. Respondents reported using a broad range of formal and informal data sources. Findings indicated differing levels of agreement on the attributes across pathway stages, with notable challenges in the *Approvals and Manufacturing* stage and consistent concerns regarding the public availability of data/evidence. The study offers valuable insights for optimising evidence generation and utilisation across the malaria diagnostic pathway. It highlights the need for enhanced stakeholder collaboration, improved data availability, and increased funding to support effective evidence generation, sharing, and use. We propose actionable solutions, including the use of public data repositories, progressive data sharing policies, open-access publishing, capacity-building initiatives, stakeholder engagement forums, and innovative funding solutions. The developed framework and study insights have broader applications, offering a model adaptable for other diseases, particularly for neglected tropical diseases, which face similar diagnostic challenges.

## Introduction

Malaria diagnostics are a key tool in controlling and eliminating malaria, a significant cause of morbidity and mortality, particularly among children in sub-Saharan Africa [1, 2]. Despite the World Health Organization’s recommendation to test all suspected malaria cases before treatment, a significant gap remains, with only half of febrile children in these regions receiving appropriate malaria testing [1]. Beyond their role in diagnosis to prevent disease progression, sensitive and affordable diagnostic tools are crucial for tracking control and elimination efforts [3]. Current diagnostic methods, primarily rapid diagnostic tests and microscopy, are essential but have limitations that impact treatment and policy decisions [2]. Rapid tests may lack accuracy due to changing parasite genetics and low-density infections, potentially resulting in false negative and false positive results [2, 4, 5]. Additionally, microscopy requires robust infrastructure and skilled technicians. To overcome the limitations of these widely used diagnostic approaches, there is an increasing need for more sensitive and specific diagnostic methods [2, 3, 6]. This necessity is driving ongoing innovation efforts. Despite progress, the urgent need for improved malaria diagnostics remains, underlining their importance in supporting effective malaria control and elimination strategies [3, 7].

In the broader context, access to high-quality diagnostics is essential for achieving universal health coverage [8]. The COVID-19 pandemic highlighted the vital role of diagnostics in healthcare, yet they often receive less attention, funding, and action compared to pharmaceuticals. This disparity is especially marked in resource-constrained settings where essential diagnostics are frequently unavailable, unaffordable, or unsuitable [9–11]. Initiatives like the WHO Essential Diagnostics List, the 2021 Lancet Commission on Diagnostics, and the 2023 World Health Assembly resolution have been established to strengthen diagnostic capacity and improve their equitable delivery [10, 12, 13]. These efforts align with the 2030 Sustainable Development Goals, specifically target 3.3, highlighting the importance of access to high-quality diagnostic tools in combating diseases like malaria [14].

The pathway from the conception of a diagnostic tool to its widespread adoption is a complex process, involving multiple stages and spanning global and national levels. The process involves a diverse range of stakeholders, including industry, governments, national and international organisations, funding bodies, regulatory authorities, researchers, healthcare providers, and end-users. Further, most malaria-endemic countries rely heavily on external funding and support for malaria control, making entities like The Global Fund to Fight AIDS, Tuberculosis and Malaria (Global Fund), the WHO, and other agencies influential in shaping the pathway [2, 15]. Each stakeholder has distinct goals and performs a range of activities to achieve these goals. Understanding and mapping this complete process is essential for guiding the development and implementation of effective malaria diagnostic tools [16]. While such pathways have been outlined for other health areas, a common framework for malaria diagnostics that captures the varied goals and tasks of all stakeholders is not readily available [16–18]. Developing this framework is not only beneficial in identifying key stakeholders but also in understanding the entire diagnostic pathway and its interconnected activities. This understanding is key to identifying potential bottlenecks, considering the sequencing of stages/activities to optimise the overall process, and ultimately improving the effectiveness and accessibility of malaria diagnostics [11, 16–19].

The malaria diagnostic pathway depends on a broad spectrum of evidence to support informed decision-making throughout the development, selection, and deployment of diagnostic tools [20]. Each stage of this pathway likely has unique evidentiary needs, influencing the types of evidence needed, its sources, and how it is used. Additionally, what constitutes appropriate evidence use may vary among stakeholders, shaped by their individual logic and goals [21]. For example, diagnostic test manufacturers might use malaria epidemiological data to guide their business strategies, while national malaria control programmes may use similar data to inform budgeting and resource allocation [16, 21].

Despite the critical role of evidence, reliable data is often limited, affecting informed decision-making across the diagnostic pathway [11]. The Lancet Commission identified this lack of data for diagnostics as a significant challenge requiring attention [10]. To address this, the Commission calls for improved data collection and more effective use of various types of evidence, including data on affordability, test performance, patient impact, as well as the broader operational structures, including workforce, infrastructure, supply chain management, and regulatory frameworks [10]. A fundamental initial step in tackling these challenges involves identifying the varied types of evidence used along the pathway. This process involves understanding how stakeholders perceive this evidence, recognising gaps, such as in availability or quality, and identifying barriers to effective evidence generation, sharing, and use.

In this paper, we address knowledge gaps by developing a malaria diagnostic pathway and examining the types and attributes of the data and evidence that inform activities across the pathway. We introduce a diagnostic framework mapping the key phases and tasks in the development and implementation of a malaria diagnostic product. This pathway builds on existing frameworks and incorporates expert insights, covering both global and national activities. We then present the results of a stakeholder survey involving key actors across the stages of the malaria pathway. Using the activities outlined in the pathway, we report on the types of evidence stakeholders use and their perceptions of its appropriateness, availability, and quality. We identify gaps in stakeholders’ abilities to generate and use evidence effectively, focusing on the barriers to accessing high-quality and relevant evidence, and propose solutions to improve evidence generation and dissemination across the malaria pathway. Our findings are intended for a broad audience, including policymakers, researchers, and industry professionals, offering insights to improve evidence use and the overall effectiveness of the malaria diagnostic pathway.

## Methods

The study has two main objectives related to improving malaria diagnosis: i) to develop a diagnostic framework for malaria by identifying and mapping key activities from product innovation to delivery, and ii) to explore the types of evidence and data that inform these activities along the pathway, and identify gaps to improve the availability and quality of this evidence.

### Objective 1: Development of a malaria diagnostic pathway

To meet the first objective, we synthesised existing frameworks with expert insights to develop a pathway specifically tailored for malaria diagnostic processes. An exploratory rapid literature review was conducted using major online databases (e.g., PubMed, Google Scholar), institutional websites, and grey literature to identify frameworks mapping key activities in disease diagnosis, from product development to delivery. The search was not limited to malaria to capture potential insights across various diseases and geographies. Key elements relevant to malaria diagnostics were compiled from these frameworks. A preliminary pathway was drafted, outlining the major stages and associated activities required to deliver a malaria diagnostic to the end-user. This draft was refined through a consultative process with the DIAGMAL consortium, a panel of malaria diagnostic experts from industry, academia, and policy across Africa and Europe. The initial draft pathway was circulated via email to the consortium for feedback and suggestions. The revised pathway was presented at a consortium meeting for collective discussion and consensus-building, leading to its finalisation.

### Objective 2: Evaluating stakeholder perceptions on data and evidence needs

The second objective focused on understanding stakeholder perceptions regarding data and evidence needs across the malaria diagnostic pathway. We define data, information, and evidence as the raw, aggregated, and analysed information used to inform the pathway’s activities and goals. A brief literature review identified key evidence attributes necessary for informed decision-making, particularly in the context of malaria diagnostics. This review led to the selection of four criteria commonly cited as important in health sector evidence use: i) a clear understanding of the evidence requirements for the task, ii) alignment of available evidence with stakeholder needs, iii) accuracy and reliability of the evidence, and iv) its public and free availability [22–25].

An online survey was designed and self-administered using Qualtrics (Provo, UT, USA) to gather insights on these criteria and the respondents’ views on evidence needs in malaria diagnostics. The survey (detailed in S1 Text) was refined and validated before launch through feedback from DIAGMAL consortium experts. Organised by the diagnostic pathway stages developed in the first part of the study, respondents first identified the stages and specific activities they were involved in within malaria diagnostics. For each selected activity, they rated their agreement with statements on the four key attributes: i) The evidence requirements to inform this activity are clear, ii) The available evidence resources meet my needs, iii) I am confident the available resources are accurate and reliable, and iv) The resources are publicly and freely available. Responses were captured on a 5-point Likert scale, ranging from ‘strongly disagree’ to ‘strongly agree’. Additionally, open-ended questions asked respondents to provide information on the types of data/information sources they use, additional evidence needs, and key challenges or potential improvements in evidence generation and use within their specific activities. The study does not extend into examining precisely how stakeholders use the evidence to inform their activities and how this influences decision-making processes.

Potential respondents included individuals from academia, policy, programmatic, industry, and healthcare involved in malaria diagnosis at any stage of the pathway. They were identified through a comprehensive search strategy including personal networks, online searches for relevant individuals at key institutions, and authors from relevant publications identified on PubMed. Two rounds of email invitations were sent in 2022 (18–29 July and 16–30 November), totalling 1,028 invitations. The survey link was anonymous, and participants were encouraged to share it. Although our identification process prioritised professionals actively involved in malaria diagnosis, there were no specific exclusion criteria.

Descriptive statistics were used to characterise the study sample and summarise the Likert-scale responses. Responses were collated for the specific activities and presented aggregated at the pathway stage level. Thematic analysis of qualitative data from open-ended questions was performed to understand stakeholders’ perspectives across the pathway. Where direct quotes a used, the role and pathway stages are included.

### Ethics statement

All respondents provided written informed consent via a mandatory electronic checkbox before accessing the survey questions. Ethical approval was obtained from the London School of Economics Research Ethics Committee (24 May 2022). The research adhered to guidelines ensuring respondent anonymity and confidentiality, with no collection of personally identifiable data.

## Results

### Development of a malaria diagnostic pathway

Our review identified several frameworks for the development and adoption of diagnostics [11, 16–19, 26–28]. These frameworks focus on different contexts such as low-resource or global settings [16, 19, 28], point-of-care diagnostics [17], and specific diseases like tuberculosis [18, 26, 27]. These typically adopt a stage-gate model, dividing the process into distinct stages with decision points that guide progression to the next phase. The frameworks are largely similar in core stages, with some variations in their depiction, including differing terminology and breakdown of certain stages. These core stages include needs assessment, feasibility, product development and validation, regulatory approval, and product launch. Frameworks tailored to low-resource settings and tuberculosis often feature specialised activities for global stakeholder engagement in technology development and rollout, such as product development partnerships, WHO prequalification, and the involvement of global market shaping actors [16, 18, 26]. S2 Text provides an overview of these identified frameworks. We did not identify a framework specifically addressing malaria diagnostics.

To address this gap, we designed a malaria diagnostic pathway, informed by these existing frameworks and expert input. Table 1 presents this pathway, featuring six major stages and 31 associated activities. Our pathway aligns with the major stages found in existing frameworks but is tailored to malaria’s epidemiological and healthcare context, incorporating activities of global stakeholders identified in frameworks focused on low-resource settings [16, 18, 26]. Additionally, our framework integrates broader health systems features, including policy activities (e.g., product selection, guideline development [16, 18, 26]), education and training [18], advocacy [16, 18], and health system and laboratory capacity development, which are not consistently found in other pathways.

**Table 1:** Malaria diagnostic pathway with associated activities by stage.

#	Stage	#	Activity
1	Needs assessment	1	Conduct needs assessment
			*(e*.*g*., *clinical requirements*, *epidemiological context*, *provider & patient needs/expectations*, *healthcare structure*, *etc)*
		2	Identify limitations of existing diagnostics
		3	Develop use cases
		4	Develop target product profile
		5	Business planning
		6	Map key stakeholders & initiate communication
			(*e*.*g*., *developers*, *country programmes and partners*, *funders [e*.*g*., *The Global Fund]*, *WHO*, *non-profit/international organisations [e*.*g*., *FIND]*, *academia)*
2	Feasibility, development, and validation	1	Establish product concept & feasibility (arrive at go/no-go decision)
		2	Develop & optimise technology
			*(Iterative process aligned with stakeholder requirements*, *user feedback*, *& laboratory/field evaluations)*
		3	Conduct laboratory validations
		4	Conduct clinical trials
			*(In intended use settings & populations)*
		5	Validation, design-lock, & transfer to manufacturing
3	Approvals and manufacturing	1	Prepare manufacturing procedures (potentially including local manufacture)
		2	Quality assurance planning
		3	Develop/update commercialisation & global access plans
		4	Obtain international regulatory approvals & registration
			*(e*.*g*., *WHO Prequalification and endorsement*, *CE certification*, *etc)*
		5	Scale-up manufacturing
4	Preparation for launch*	1	Global endorsement, policy, & guidelines for intended use
		2	Develop supply chain & distribution plans
		3	Global plans for pricing, financing, procurement, & resource mobilisation
		4	Conduct in-country demonstration projects
			*(e*.*g*., *test performance*, *feasibility*, *implementation & scale-up barriers*, *patient/population impact*, *& cost-effectiveness)*
5	Adoption and scale-up*	1	Obtain local regulatory approvals & registrations
		2	Develop national policy to support optimal selection & delivery of diagnostics
			*(e*.*g*., *selection of diagnostics*, *policy and guideline development*, *etc)*
		3	Develop & execute implementation and scale-up strategy
			*(e*.*g*., *selection of sites*, *delivery channels*, *workflow integrations*, *etc)*
		4	Country plans and policy for budgeting/financing
		5	National/local procurement
		6	On-the-ground advocacy efforts to ensure technology adoption
			*(e*.*g*., *healthcare provider & end-user education*, *training*, *civil society engagement)*
		7	Health system and laboratory strengthening to support delivery (infrastructure & human resources)
6	Surveillance and impact measurement	1	Monitor implementation quality & address field issues
		2	Optimise delivery strategies through operational & policy research
		3	Robust health impact assessment
			*(e*.*g*., *increased case detection*, *therapeutic outcomes*, *lives saved/DALYs*, *equity*, *etc)*
		4	Economic evaluation
			*(e*.*g*., *healthcare costs*, *benefits*, *& affordability)*
		5	Post-marketing surveillance

Abbreviations: CE, Conformité Européenne; DALY, disability-adjusted life year; FIND, Foundation for Innovative New Diagnostics; WHO, World Health Organization

*Key activities within these stages may occur in parallel or overlap to increase efficiency.

The pathway begins with the *Needs assessment* stage, establishing technology requirements and stakeholder engagement, forming the foundation for subsequent stages. Based on these findings, *Feasibility*, *development*, *and validation* follows, involving an iterative process to develop and optimise the technology, aligning with stakeholder needs and feedback. This is followed by the *Approvals and manufacturing* stage, focusing on international regulatory approvals, including WHO prequalification, and production scale-up. *Preparation for launch* involves global activities to support uptake of the tool, running parallel to in-country demonstration projects. *Adoption and scale-up* is aimed at integrating the tool effectively into healthcare systems. Finally, the *Surveillance and impact measurement* stage monitors implementation quality and optimises delivery strategies. While presented sequentially, the process is cyclic and iterative in practice, reflecting a continuous process of evaluation, adaption, and improvement. To increase efficiency, certain stages, like *Preparation for launch*, should ideally overlap and run concurrently with others, such as *Adoption and scale-up*, ensuring rapid and effective implementation.

### Stakeholder perspectives on data and evidence needs

A total of 113 individuals consented to participate in the survey (Table 2). Respondents were mainly active in the Africa region and spanned a range of sectors, including academia, non-governmental organisations, international organisations, governmental bodies, healthcare professions, and industry representatives. Academia had a strong representation, accounting for 39% of respondents. All stages of the diagnosis pathway were represented in the sample, with most respondents involved in activities in more than one stage.

**Table 2:** Characteristics of the survey respondents (n = 113).

	*N*	*%*
	113	
*Geographical focus of work (WHO regions)*		
Africa Region	71	63%
South-East Asia Region	28	25%
Western Pacific Region	9	8%
Region of the Americas	8	7%
European Region	8	7%
Eastern Mediterranean Region	4	4%
Missing	21	19%
*Role (current or most recent employment)*		
Academic group	44	39%
International NGO	21	19%
Government	18	16%
Clinician or other healthcare professional	16	14%
Implementation/technical assistance partner	13	12%
Laboratory scientist	13	12%
Product development partnership	5	4%
Industry/diagnostic manufacturer	5	4%
Donor/funding agency	3	3%
Advocacy group	2	2%
Regulatory agency	1	1%
National NGO / CSO	1	1%
Missing	21	19%
*Stages of the continuum involved in* *		
Needs assessment	43 (119)	38%
Feasibility, development, & validation	55 (121)	49%
Approvals & manufacturing	10 (19)	9%
Global preparation for launch	12 (26)	11%
Country adoption & scale-up	34 (82)	30%
Surveillance & impact measurement	56 (83)	50%

Answers do not sum to *N* as respondents could tick more than one response.

Country was optional and answered by ~50% of respondents, so is not presented. The 53 respondents answering this question represented 28 countries.

*Presented as *N (n)*, where *N* = number of respondents and *n* = total number of activities respondents indicated they were involved in. In addition to the activities presented in Table 1, for each stage, respondents were able to select “other” & specify an activity.

Respondents utilised a wide range of data and evidence sources, from local context-specific data to global reports (Table 3). Both generic and specific sources were highlighted, with many being utilised across multiple stages. Commonly cited evidence types included epidemiological data, routine and operational data on case and testing numbers, health facility surveys, effectiveness and cost-effectiveness data, national plans, World Malaria Reports, and other global reports and guidelines (e.g., from the Global Fund, the Foundation for Innovative New Diagnostics [FIND], and WHO). Certain sources were identified as more stage-specific, such as WHO and FIND target product profiles (TPPs) (*Needs assessment* and *Feasibility*, *development*, *and validation*), manufacturer’s cost of goods data (*Approvals and manufacturing*), and pharmacovigilance data (*Surveillance and impact measurement*). Data sources included published literature, Health Management Information Systems [HMIS], health ministries and national malaria programmes, global organisations, and research institutions. In addition to publicly available data, many respondents also relied on self-generated data and significantly on informal channels of information, such as feedback and evidence shared through conversations, meetings, and discussions with colleagues, experts, donors, regulatory authorities, and global organisations (e.g., Global Fund and WHO).

**Table 3:** Types of evidence used across the malaria diagnostic pathway.

Pathway stage	Forms/type of evidence	Source of evidence
1) Needs assessment	Epidemiological data and maps World Malaria Reports Effectiveness, cost-effectiveness, and specifications of existing tools TPPs Market research (e.g., market size) Routine and operational data (e.g., case number, testing numbers, etc) Global and local policy documents	Published literature (e.g., journal articles, conferences) MoH/NMCP of target countries Global organisations (e.g., WHO, FIND) Surveys and routine data sources (e.g., HMIS, LMIS, health facilities) Researchers/research centres/institutions (national and international) Self-generated data Informal channels (e.g., unpublished data, discussions)
2) Feasibility, development, and validation	TPPs Laboratory results Clinical trial data and field evaluations User feedback Guidelines (e.g., REASSURED and STARD)	Published literature Product developers/manufacturers (including confidential data) Global organisations (e.g., WHO, FIND) Self-generated data
3) Approvals and manufacturing	Manufacturer’s cost of goods data Product specifications Manufacturer records Effectiveness and cost-effectiveness data Global plans and strategies	Product developers/manufacturers Data consultants Regulatory bodies Global organisations (e.g., product development partnerships, donors, etc)
4) Preparation for launch	Routine and operational data (e.g., number cases and tests, facility surveys, pilot studies, etc) Expected and final budgets (global and national) Supply chain and procurement analyses Strategy and planning documents	MoH/NMCP/MoF TWGs Global organisations (e.g., WHO, Global Fund) Regulatory bodies Surveys and local routine data sources (e.g., HMIS, LMIS, health facilities) Informal channels (e.g., meetings and discussions)
5) Adoption and scale-up	Effectiveness and cost-effectiveness data Demonstration project findings Epidemiological data (local/sub-national) Modelling data Routine and operational data (e.g., basic information on what is being done and where, commodity/stock data, health facility mapping, etc) Global policy documents (e.g., guidelines, WHO prequalification, syntheses of strategies) National plans, guidelines, budgets	Published literature (e.g., journal articles, conference proceedings, meetings) MoH/NMCP/MoF and other ministerial departments TWGs Global organisations (e.g., WHO, Global Fund) Surveys and local routine data sources (e.g., HMIS, LMIS, health facilities) Researchers/research centres (national and international) Informal channels (e.g., meetings and discussions)
6) Surveillance and impact measurement	Pharmacovigilance and surveillance data Health impact and costing models/analyses Patient/provider experiences Routine and operational data (e.g., tool usage and coverage, performance metrics, etc) Data on implementation challenges (e.g., supply chain, workforce training, workflow)	MoH/NMCP Researchers/research centres (national and international) Surveys and local routine data sources (e.g., HMIS, LMIS, health facilities)

Abbreviations: FIND, Foundation for Innovative New Diagnostics; HMIS, Health Management Information Systems; LMIS, Logistics Management Information Systems; MoH, Ministry of Finance; MoH, Ministry of Health; NMCP, National Malaria Control Programmes; STARD, Standards for Reporting for Diagnostic Accuracy Studies; TPP, Target Product Profiles; TWG, Technical Working Group; WHO, World Health Organization

Note: The table is not designed to be an exhaustive mapping, but summarises the key forms and sources of evidence described by the survey respondents

**Fig 1:**
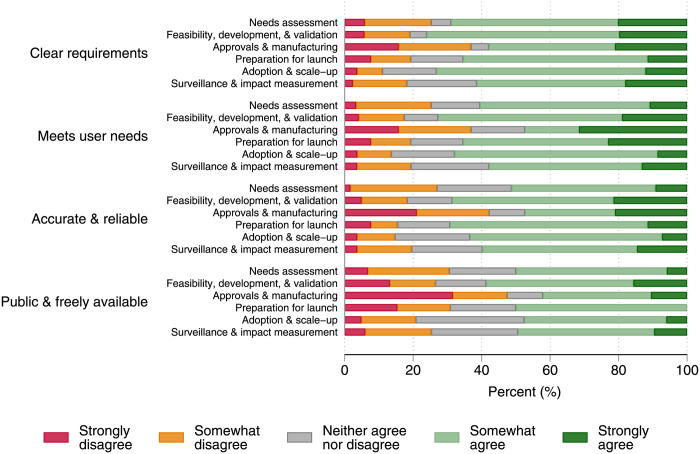
Survey findings for the four key attributes across the different pathway stages.

Fig 1 presents the respondents’ perceptions of the four evidence attributes across different stages of the malaria diagnostic pathway. Overall, most respondents believed the evidence requirements for their activities were clear, with 58%-76% somewhat or strongly agreeing across the stages. The survey also indicated that most respondents believe the available data and evidence resources met their needs (47%-73% across the stages) and were accurate and reliable (47%-69%).

The *Approvals and manufacturing* stage consistently showed higher levels of disagreement across all attributes, with 37%-47% of respondents indicating some level of disagreement, though this stage had a smaller sample size (n = 10 respondents). Conversely, evidence attributes for stages including *Needs assessment*, *Feasibility*, *development*, *and validation*, *Preparation for launch*, and *Adoption and scale-up* were more favourably perceived.

A consistent area of concern across all stages was the public and free availability of data. Between 21%-47% of respondents across the stages at least somewhat disagreed with this aspect. Data from Fig 1 is available in S3 Text.

Respondents identified several challenges and opportunities related to effective generation, sharing, and application of data and evidence across the malaria diagnostic pathway (Table 4). These were classified into four broad areas.

**Table 4:** Summary of challenges and opportunities to improve the availability of evidence across the malaria diagnostic pathway.

Theme	Challenge	Potential solutions & opportunities
Stakeholder collaboration	Poor coordination among stakeholders	Develop forums or platforms that allow stakeholders to openly share plans, encourage feedback, share experiences, and identify/address key data and evidence gaps Establish a single online location where all resources relevant to the pathway are stored and linked (e.g., TB diagnostics critical pathway website) Integrate stakeholders, particularly National Malaria Control Programs and other key players, from early-stage research to ensure tools are appropriate, useful, and aligned with global/national efforts Implement mechanisms to speed up the translation of evidence from the global to the national level to efficiently inform national policy Encourage formal and informal meetings, conversations, and exchanges between stakeholders to share (and request) evidence
	Needs and opinions of users and patients not sufficiently addressed	Conduct user experience studies Establish regular channels of communication and collaboration between end-users, patients, and developers at every stage of the pathway Establish funding to facilitate end-user research
Data availability	Poor availability, quality, and timeliness of data	Invest in capacity building to improve robust data collection, analysis, and use, particularly at the operational level Leverage digital health information systems (e.g., HMIS and LMIS) to streamline data collection processes and ensure relevant indicators are captured Implement routine data quality assessments Develop mechanisms for rapid data sharing and dissemination of operational data to inform the development of new tools Take advantage of new opportunities (such as new local development and manufacturing hubs) to collect targeted data on end-user populations
	Incomplete availability and publishing of data and datasets and lack of transparency	Encourage and support open-access publishing and licensing of findings Promote the use of public data repositories and registries for data/datasets/resources (e.g., Figshare, Harvard Dataverse, Clinicaltrials.gov, etc) Implement progressive data management and sharing policies (e.g., data sharing as a requirement for funding or collaboration) Promote open data sources to provide reliable and comparable data (e.g., Malaria Atlas Project, PlasmoDB, etc) Make additional efforts to ensure key missing data are available and open source (e.g., key data from manufacturers and regulators for the Approval and Manufacturing stage) Promote prospective registration of diagnostic trials Prioritise the dissemination of findings from poorly performing tests to enhance lessons learned
Standardisation	Lack of standardisation of protocols	Develop and disseminate standardised protocols and guidelines for evaluation of diagnostics Organise training sessions to educate stakeholders on standardised procedures Collaborate with international organisations to align protocols with global best practices
Funding	Lack of funding support	Advocate for increased funding from diverse sources including governments, international organizations, and private donors Establish dedicated funding mechanisms for the malaria diagnostic research underpinning each of the pathway stages Explore innovative financing models and public-private partnerships

The solutions identified in the table are a mix of those identified by stakeholders in the survey and those identified through a review of the literature.

#### 1) Stakeholder collaboration

A common theme among respondents across all stages was the desire for better data and evidence sharing, cooperation, and communication among stakeholders. For instance, one respondent stressed that “*country partners implementing these strategies must communicate and share experiences regularly*” [government; stages 1,5,6]. Another voiced frustration about researchers not adequately engaging with national malaria programmes: “*too many rich world based researchers who don’t spend enough time working day-by-day with national malaria programs to develop findings that are actually used*” [product development partnership/implementation partner; stages 2–6]. Poor coordination was reported to lead to siloed data, lack of cohesive action, evidence gaps, and missed opportunities for data integration into decision-making processes. To address this, respondents advocated for mechanisms to improve collaboration for early stages of diagnostic development, with one respondent recommending the creation of “*a forum where all or most stakeholders are able to openly share plans*” [government; stages 1,2,5,6].

Many respondents underscored the need for greater emphasis on patients’ and end-users’ needs and perspectives across all stages during the development and implementation of diagnostic tools. They identified that ineffective collaboration with these groups hinders the collection and utilisation of essential data. One respondent advocated for improved dialogue with intended users, stating: “*more conversations with intended users and patients need to be brought into every stage of the process*, *but it’s hard to get donors to fund this*” [implementation partner; stages 2–6]. Another sought “*clearer information on user needs and expectations”* [academic; stages 1,2,6], to better integrate these considerations into the diagnostic development process.

#### 2) Data availability

Several respondents emphasised the need for more, and better quality, data to inform their activities across the malaria diagnostic pathway. They expressed concern over the lack of available data necessary for certain activities or decisions: “*often data required along the pathway are not available and need to be collected before certain activities can start or decisions made”* [government; stages 1,5,6]. Respondents called for more reliable evidence on a range of aspects including local epidemiology, pricing, procurement, test consumption, scientific experimental data, pharmacovigilance, and program/intervention costs and cost-effectiveness.

The need for more detailed data was also emphasised, evident in one respondent’s wish for “*more data at facility or CHW [community health worker] level; what do they see as shortcomings or limitations of the tools available*” [international NGO; stages 1,2,3,5,6]. The capacity to generate operational and technical data was also a concern: “*economic evaluations are a serious handicap in evidence to drive policy … capacity in operational research is also needed*” [implementation partner/laboratory scientist; stages 5,6], suggesting the need for investments in capacity building for data collection, analysis, and use.

There was a call for better public availability and sharing of data and datasets from routine and research activities, with solutions including open-access publishing and the use of data repositories. Respondents suggested that such data could inform activities directly or be used secondarily to generate further evidence. For available data, they desired a better understanding of limitations: “*for each data source*, *sufficient information to determine limitations of methods used to generate the data*” [government; stages 1,5,6]. Calls for greater transparency were also evident, with requests for insight into failed tests, diagnostic trials, industry secrets, and access to unpublished data. One respondent recommended a “*single virtual location where all these info on the pathways and how to implement them are situated*” [international NGO/academic/laboratory scientist; stages 1,2,3,6].

Finally, some respondents reported challenges concerning the timeliness of data and evidence for decision-making and called for mechanisms to improve evidence sharing and dissemination. Specific issues mentioned included slow regulatory processes and delayed transfer of global evidence to the national level, for example, “*it often takes many years before signals of diagnostic failure to change policy and the introduction of new tools is a long and laborious process*” [international NGO/healthcare professional; stages 1,2,5,6].

#### 3) Standardisation

Participants reported issues related to standardisation of protocols and data requirements, particularly in the context of laboratory validations and clinical trials (i.e., stage 2: feasibility, development, and validation). For instance, one respondent stated: “*no standardised protocols for field evaluation of diagnostics (e*.*g*., *level of blood screen by PCR) exist*” [academic; stages 1,2,6]. They also underscored the need for clarity about the aspects of the test that need characterisation and the precise data and evidence required, especially data crucial for regulatory approvals.

#### 4) Funding

Funding constraints was frequently highlighted as a significant barrier, impacting both the generation and analysis of key data for each step on the malaria diagnostics development pathway, as well as the dissemination and utilisation of evidence-based practices. Respondents discussed the difficulty of securing funding for basic research: “*funding for basic research which underpins each step is not available*. *There is no funding for basic research on diagnostics that I can secure or even apply for*” [academic; stages 1,2] and advocated for increased funding from a range of sources.

Of note, while many respondents highlighted challenges, a few commented that the data available for malaria was already sufficient for their purposes and emphasised that *“malaria diagnostics is much better documented than most other diseases*, *other than HIV and tuberculosis”* [academic; stage 4].

## Discussion

This study sought to map the malaria diagnostic pathway, highlighting key data and evidence needs at each stage and stakeholder perceptions of this evidence. While the main stages of our developed pathway echo existing frameworks, a malaria-specific pathway offers distinct advantages by integrating activities tailored to the specific challenges and context of malaria diagnosis [11, 16–19]. For instance, it integrates priority activities like WHO prequalification and endorsement processes–pivotal given the limited or complex diagnostics regulatory frameworks in many resource-constrained settings [16, 29]. It also promotes stakeholder collaboration, promoting better alignment with global and national malaria eradication and control efforts, and engages key players, like The Global Fund, WHO, and FIND, who provide vital financial, technical, and strategic support [16]. By focusing on malaria, the framework structures the interconnected activities essential to the malaria diagnostic process, serving as a targeted tool for planning, coordination, and action across different stages and sectors.

Expanding beyond malaria, our framework has broader potential applicability, especially for neglected tropical diseases (NTDs) and other high-priority pathogens with critical diagnostic challenges (e.g., Chagas disease, leishmaniasis, lymphatic filariasis, etc) [30–33]. The overarching challenges our study highlights, such as data scarcity and funding issues, are not exclusive to malaria and may be amplified for NTDs due to their limited attention and resources compared to the “big three” infectious diseases [34, 35].

The survey identified both strengths and weaknesses in data and evidence along the malaria diagnostic pathway. Respondents’ perceptions of the evidence were largely positive during stages like *Feasibility*, *development*, *and validation*, and they emphasised the good availability of evidence for malaria diagnostics compared to other diseases. The *Approvals and manufacturing* stage emerged as particularly challenging, with the highest rates of disagreement across all data attributes. Factors potentially contributing to this are complex regulatory processes and the reliance on confidential and proprietary information during this stage [36]. To addresses these issues, strategies such as financial incentives (e.g., grants), non-financial incentives (e.g., industry recognition or priority status in regulatory processes), or standardised data sharing agreements might be needed to improve data availability and transparency during this stage.

Addressing the key data challenges identified requires innovative solutions and concerted efforts across multiple sectors. Potential solutions, identified by stakeholders and the broader literature, are presented in Table 4 and include enhanced stakeholder coordination, improvements in data generation, availability, and sharing, and improved sustainable funding.

Effective stakeholder coordination from early development phases can bridge knowledge gaps, foster data sharing, and facilitate more informed decision-making, thereby accelerating diagnostics development and use. Bridging collaboration gaps can be achieved through **forums or strategic partnerships** focused on sharing plans, seeking feedback, and addressing evidence gaps. The GeneXpert TB test development and the Digital Diagnostics for Africa Network are examples of successful cross-collaboration models that bring together stakeholders from different fields and pathway stages to support evidence generation and sharing [37–39]. Additionally, a **centralised resource hub for malaria diagnostics** could improve transparency and collaboration by centralising research, guidelines, and data sources. The TB Diagnostics Critical Pathway website serves as an analogous model, illustrating the TB pathway and consolidating relevant resources, data, and evidence for each phase [18]. Beyond formal structures, our study highlights the important of **informal networks and trusted personal collaborations** as effective communication and evidence exchange channels, as observed in other health sector studies [40, 41]. Encouraging meetings, conferences, and reflective spaces for individuals at different stages of the pathway can serve as effective mechanisms for providing or requesting evidence.

Gaps in high-quality data can be mitigated by investing in **capacity-building initiatives for data collection, analysis, and utilisation**. A key intervention would be promoting the widespread adoption and improved use of routine electronic HMIS to obtain quality and timely real-world health and operational data. Investments in these systems, as well as the supporting associated infrastructure and personnel, can deliver comprehensive data on disease burden, service utilisation, and test performance, informing the targeted development and implementation of diagnostic tools tailored to the population and health system’s specific needs [10]. The District Health Information Software (DHIS2) platform serves as an exemplar, widely adopted in many countries [24]. Further, as diagnostic research, development, and manufacturing hubs gradually expand to include more middle- and low- income settings, there are new opportunities to ensure that the data collected on the diagnostic pathway reflect the needs of the target populations the diagnostics are designed to serve [42].

Promoting data and evidence sharing can support accessibility, enable transparency and validation of findings, and encourage the reuse of data for further research [43, 44]. Strategies include **promoting open-access publishing** and the use of **online data repositories and registries** (e.g., Figshare or Harvard Dataverse) [20, 43]. These efforts should be accompanied by **progressive data management and sharing policies** [45]. Recent advancements in data sharing policies, with mandates on using public data repositories for publication in certain journals and funding opportunities dependent on open-access publishing and a commitment to data sharing, highlight progress in this area [43, 46, 47]. However, these mechanisms remain underutilised. Concerns over loss of exclusivity, fears of challenging interpretations, data protection issues, and data formatting hurdles may discourage researchers from sharing data [43, 48]. Collective efforts from funders, industry, research institutions, researchers, and publishers are required to encourage an ecosystem where open and free data access is not only feasible but actively encouraged and rewarded.

Central to data sharing are efforts to champion diverse open-access resources, like the Global Health Data Exchange (for disease burden), the Malaria Atlas Project (for malaria epidemiology), PlasmoDB (for plasmodium informatics), and G-FINDER (for research and development investments) [49–52]. **Expanding these open data initiatives** across the diagnostic pathway could substantially improve data accessibility and ensure an evidence-based approach to diagnostic development. While there is consensus on the value of an open approach to data sharing, the extent to which the malaria community should leverage generalised platforms or develop malaria-specific ones remains a topic of discussion. We propose that a broader, more inclusive, effort would gain more traction.

Notably, available diagnostic databases frequently lack the comprehensiveness of their pharmaceutical counterparts. For example, while clinical trials registries provide comprehensive details on pharmaceutical trials, diagnostic accuracy studies are rarely registered [53]. Advocating for the **mandatory, prospective registration of diagnostic trials** can support a more thorough understanding of diagnostic efforts, amplifying transparency and reporting, and crucially encouraging the transparent reporting of negative results. Prioritising access to relevant evidence across the entire pathway, including data not typically published in journals and less favourable findings, is essential for improving accessibility and promoting an evidence-based approach to malaria diagnosis.

Inadequate funding also emerged as a key challenge, restricting essential data collection, analysis, and dissemination [10, 54, 55]. This challenge is accentuated for diseases like malaria and many NTDs that primarily affect lower-income settings. In these contexts, the demand for tailored diagnostic solutions–such as affordable rapid tests rather than cost-intensive platforms–is pressing, yet the modest market size often deters commercial diagnostic manufacturers from investing. Addressing this challenge necessitates **advocacy for increased and diverse funding sources**, including from donors, international organisation, and governments, specifically targeting the development, implementation, and evaluation of appropriate diagnostic tools for resource-constrained settings and data and evidence generation, sharing, and utilisation along the pathway. Strategies like dedicated funding mechanisms, innovative financing models, and public-private partnerships can incentivise stakeholders to contribute to the data ecosystem and overcome these challenges. For instance, prize funds for diagnostic data generation, similar to those that incentivise diagnostic development, offer a potential model [56].

While the study provides valuable insights, certain limitations should be considered when interpreting the findings. First, the survey could be subject to response bias, and its low response rate with overrepresentation from academia may not capture the full range of perspectives and experiences in the global malaria diagnostic landscape. Stages with fewer respondents, such as *Approvals and manufacturing* and *Global preparation for launch*, may be particularly susceptible to bias; further perspectives from stakeholders involved in these stages would be beneficial. The study was part of an explorative process and was not expected to represent an exhaustive summary of all data challenges. Second, the self-reported nature of the data could lead to biases like recall bias. Finally, focusing on a malaria-specific pathway may limit the generalisability of our findings across other diseases. Notably, our findings align to those suggested by an online expert panel in a study on global health diagnostic solutions, suggesting broader applicability of our findings [17]. The study did not investigate exactly how stakeholders use different types of evidence to inform decisions and activities, nor did it explore ways to optimise this, which represent key areas for future research, as seen in other malaria and health policy studies [21, 57–60].

This study offers valuable insights into the malaria diagnostic pathway, highlighting key challenges and direction for future efforts to improve the scope and availability of quality data and evidence across the pathway. Strengthening the generation and use of data and evidence to inform diagnostic development and policy requires a multifaceted approach, focused on improving the availability and accessibility of diverse and rich data, supported by effective stakeholder collaboration and sustainable funding. Overcoming these challenges and improving access to more diverse evidence forms is increasingly important as new and more innovative diagnostic tools are developed. Leveraging frameworks like the one proposed in this study can help identify data gaps and guide the evidence-based innovation and implementation of diagnostic tools. The framework and challenges identified has implications not only for malaria but can also serve as a model to be adapted for other diseases, especially NTDs, which face similar, if not more significant challenges.

## Supporting information

S1 Text Malaria diagnostic pathway data and information survey. (DOCX)

S2 Text Key characteristics of the existing diagnostic frameworks. (DOCX)

S3 Text Data and ranking for Fig 1. (DOCX)
