# Supplementary material for: Defining a malaria diagnostic pathway from innovation to adoption: Stakeholder perspectives on data and evidence gaps
Source: PLOS Glob Public Health. 2024 May 16;4(5):e0002957. doi: 10.1371/journal.pgph.0002957 (PMC11098419; doi:10.1371/journal.pgph.0002957)
Supplement: S1 Text — (DOCX) [file pgph.0002957.s001.docx]

**S1 Text. Malaria diagnostic pathway data and information survey**

Start of Block: Introduction

**Introduction**

Ensuring access to effective diagnosis of disease is a complex process involving several stages from product design and innovation through to successful adoption. In this study we focus on malaria diagnosis. We define this continuum of diagnosis using six key phases^1^ as follows:

1. Needs assessment
2. Feasibility, development, & validation
3. Approvals & manufacturing
4. Global preparation for launch
5. Country adoption & scale-up
6. Surveillance & impact measurement

Each stage consists of several activities (shown here). Key challenges to the supply and demand of diagnostics exist across all stages and activities of the continuum. These challenges are further compounded by a lack of robust data, information, and evidence to support activities and inform decisions.

The purpose of this survey is to understand what data and information are used to inform activities, decision-making, and policy across the difference stages of the continuum. We seek to understand whether the available data meets users’ needs and what additional data is required.

Thank you for your participation.

*^1^These stages are informed by* [Mugambi et al. 2018](https://gh.bmj.com/content/3/6/e000914) *(BMJ Global Health),* [Engel et al. 2016](https://gh.bmj.com/content/1/4/e000132) *(BMJ Global Health), and the* [TB diagnostics critical pathway](https://www.tbdxpathway.org/) *2017 (Research Institute of the McGill University Health Centre)*

End of Block: Introduction

Start of Block: Informed Consent

**Informed consent**

You have been invited to take part in this study to gain insight into data availability and use along the malaria diagnosis continuum. The study will be conducted by researchers at the London School of Economics & Political Science (LSE).

If you agree to take part, you will be asked to take part in an online survey. The survey consists of questions about the specific activities you are involved in and the data/information you use, as well as a few questions about you.

It should take about 10-15 minutes to complete.

Participation is entirely voluntary. Your responses will be confidential, and all data will be securely stored. We will use the collected information for a research project that may result in publication.

The study has undergone ethics review in accordance with the LSE Research Ethics Policy and Procedure.

If you have any questions regarding this study please contact the researcher, Dr Bryony Simmons, on [b.n.simmons@lse.ac.uk](mailto:b.n.simmons@lse.ac.uk). If you have any concerns or complaints regarding the conduct of this research, please contact the LSE Research Governance Manager via [research.ethics@lse.ac.uk](mailto:research.ethics@lse.ac.uk).

Are you willing to participate? If you choose not to participate, the survey will end immediately.

- Yes
- No (stop survey)

Skip To: End of Survey If Informed consent = No (stop survey)

End of Block: Informed Consent

Start of Block: Section 1: Respondent characteristics

**Section 1: Respondent characteristics**

1. In which country are you based? [dropdown menu with list of countries]

▼ Afghanistan ... Zimbabwe

1. Which WHO region best describes the focus of your work? (select all that apply)
   - Africa Region
   - South-East Asian Region
   - Eastern Mediterranean Region
   - Region of the Americas
   - Western Pacific Region
   - European Region
2. Which type of role best describes your current or most recent employment? (select all that apply)
   - Government (please specify body/department): ___________________________________
   - Regulatory agency
   - Donor/funding agency
   - Product development partnership
   - Implementation/technical assistance partner
   - International NGO
   - National NGO / CSO
   - Academic group
   - Laboratory scientist
   - Clinician or other healthcare professional
   - Advocacy group
   - Industry/diagnostic manufacturer
   - Other (please specify): ___________________________________
3. Relating to malaria diagnosis and control, are you involved in any of the following? (select all that apply)
   - Policy decisions & activities
   - Programmatic decisions & activities
   - Data users (eg, program managers, clinical staff, etc)
   - Data producers (eg, M&E personnel, academic groups, etc)
   - Product innovation & manufacture

[Options: Subnational/local; National; International/global]

End of Block: Section 1: Respondent characteristics

Start of Block: Section 2: Diagnosis continuum, evidence use and generation

**Section 2: Diagnosis continuum, data and information use and gaps**

We define the diagnosis continuum using the following six key stages:

| **Continuum stage** | | **Description** |
| --- | --- | --- |
| **1** | **Needs assessment** | Assess the extent of the problem & develop target product profile/use cases |
| **2** | **Feasibility, development, & validation** | Determine whether a product can be developed to meet user needs, develop the technology, & conduct laboratory & field evaluations |
| **3** | **Approvals & manufacturing** | Obtain international approvals & develop & execute manufacturing procedures |
| **4** | **Global preparation for launch** | Develop global policy, pricing, procurement, & supply chain plans, & carry out large-scale demonstration projects |
| **5** | **Country adoption & scale-up** | Develop national policy & implement rollout & scale-up plans |
| **6** | **Surveillance & impact measurement** | Measure the ability of the test to improve outcomes for the patient & the impact on population health & conduct post-marketing activities |

*Note: While depicted as a linear process from inception to adoption, the process is likely to be iterative. Additionally, stages can occur in parallel or overlap.*

1. Which of these stages are you involved in in your current role? (select all that apply)
   - Needs assessment
   - Feasibility, development, & validation
   - Approvals & manufacturing
   - Global preparation for launch
   - Country adoption & scale-up
   - Surveillance & impact measurement

Skip to: Block on specific continuum stage If Stage = selected

(Based on the responses to this question, the following sections will display for each of the continuum stages that the respondent indicates they are involved in)

End of Block: Section 2: Diagnosis continuum, evidence use and generation

Start of Block: <Continuum stage>

**<Continuum stage>**

1. For this continuum stage, what specific activities are you involved in? (select all that apply)
   - *Populated by column 2 of table on page 1 (for the specified stage)*

Skip to: Block on specific activity if Activity = selected

(The following block will display for each of the activities the respondent indicates they are involved in. The questions will appear for each activity distinctly. The list of activities by stage is found on page 1)

End of Block: <Continuum stage>

Start of Block: <Continuum stage>: <Activity>

**<Continuum stage>: <Activity>**

*In this section, please consider data/evidence resources related to the specified activity only. There will be a chance at the end of the survey to mention any other resources.*

1. For this activity, how strongly do you agree with the following statements:
   1. The evidence requirements to inform this activity are clear
   2. The available data/evidence resources meet my needs
   3. I am confident the available resources are accurate & reliable
   4. The resources are publicly & freely available

I rely on the following types of resources:

- 1. Subnational/local data
  2. National data
  3. Global/international data

[Options: Strongly disagree; Somewhat disagree; Neither agree nor disagree; Somewhat agree; Strongly agree]

1. (Optional) What data/evidence resources do you use for this activity?

[string response]

1. (Optional) What additional data/evidence resources do you think are needed for this activity?

[string response]

1. (Optional) In your opinion, what are the key challenges relating to data and evidence for this activity?

[string response]

End of Block: <Continuum stage>: <Activity>

Start of Block: Final remarks

**Final remarks (optional)**

1. Beyond the data/evidence you have already listed, are there any additional key sources across the continuum that you would like to identify?

[string response]

1. Beyond the data/evidence gaps that you have already listed, are there any additional key knowledge gaps across the continuum that you would like to identify?

[string response]

End of Block: Final remarks
