# Supplementary material for: Defining a malaria diagnostic pathway from innovation to adoption: Stakeholder perspectives on data and evidence gaps
Source: PLOS Glob Public Health. 2024 May 16;4(5):e0002957. doi: 10.1371/journal.pgph.0002957 (PMC11098419; doi:10.1371/journal.pgph.0002957)
Supplement: S2 Text — (DOCX) [file pgph.0002957.s002.docx]

**S1 Table. Key characteristics of the existing diagnostic frameworks**

| **Reference** | **Framework application** | **Main stages** | **Framework highlights** | **Relevance to malaria pathway and data** |
| --- | --- | --- | --- | --- |
| **World Economic Forum** *(2021)* [1] | Global | 1. Concept 2. Feasibility 3. Development 4. Verification 5. Validation | Provides a generalised skeleton pathway to support a wider discussion on global implementation of diagnostics. | Highlights data at every level as a crucial driver of diagnostic development and implementation: “*data is the foundational unit driving diagnostic development and implementation*”. |
| **Mugambi et al.** *(2018)* [2] | Low-resource settings | 1. Concept and research 2. Product feasibility, definition and planning 3. Design, development and transfer to manufacturing 4. Validation, regulatory approvals and first launch 5. Post-launch surveillance and stable operations | Detailed description of activities from discovery through delivery, segregated by type: i) marketing and sales activities, ii) R&D, manufacturing, regulatory and legal activities, and iii) policy and advocacy activities. | Highlights specific activities characteristic of LMICs involving global stakeholders (e.g., supporting inclusion of diagnostic product in WHO recommendation, WHO prequalification, developing policymaker and funder engagement strategies, developing global access strategies, etc). Includes policy and advocacy activities. |
| **Derda et al.** *(2015)* [3] | Point-of-care diagnostics / low resource settings | 1. Demand and initial assessment 2. Research and development 3. Evaluation and adaptation 4. Regulation via national & international bodies 5. Production QA/QC. Delivery support | Value chain developed during a workshop on POC diagnostics (held in Kenya, 2012). Used to guide discussion on barriers to the successful development and distribution of POCs. | Similar to our study, highlights gaps in needs assessment and stakeholder flow of information as challenges along the chain. |
| **Engel et al.** *(2016)* [4] | LMICs / point-of-care diagnostics | 1. Demand and initial assessment 2. Research and development 3. Evaluation and adaptation 4. Regulation via national & international bodies 5. Production QA/QC. Delivery support | Applies Derda et al.’s framework, incorporating insights from a global health discussion platform on factors driving demand and supply for diagnostics. | Emphasises research and evidence generation as key barriers along the diagnostic value chain, echoing our survey findings (specifically: lack of end-user engagement, limited stakeholder engagement, lack of local multidisciplinary research capacity, and insufficient evaluation of diagnostics). |
| **Research Institute of the McGill University Health Centre** *(2017)* [5] | Tuberculosis | 1. Identify need 2. Concept and feasibility 3. Development 4. Lab and clinical validation 5. Regulatory approvals 6. WHO evaluation 7. Country transition to scale (pre-launch) 8. Country adoption 9. Scale-up and monitoring 10. Impact measurement | Provides a granular TB diagnostics pathway, hosted on a website bringing together resources and information to support the rapid introduction and widespread scale-up of new TB tools. | Highlights global activities necessary for TB, including WHO evaluation, global policy, and global procurement.  The pathway also incorporates wider health systems attributes for successful adoption, such as training, advocacy, and civil society engagement.  Online hub for TB resources as recommended in our survey that might be useful for malaria. |
| **World Health Organization** *(2009)* [6] | Tuberculosis | 1. Needs assessment 2. Concept, feasibility and proof-of-principle 3. Development, optimization (design-lock) 4. Evaluation studies of accuracy and reliability 5. Demonstration studies including patient outcomes 6. Evidence for scale-up, scale-up, delivery and access 7. Policy 8. Epidemiological and public health impact | Serves as a blueprint for developing TB diagnostics. Outlines the appropriate value chain for the development of new diagnostics to be submitted for scientific review and endorsement to WHO, and to inform global policy and development. | Focus on global policy and regulatory activities necessary for TB.  Emphasises the need for data and evidence at each stage. The report lists key data elements needed at each stage (e.g., epidemiological context, user requirements, test performance data) and details some data sources. It proposes a framework for collating evidence in impact assessment. |
| **Albert et al.** *(2016)* [7] | Tuberculosis (specifically Xpert MTB/RIF) | 1. Product development 2. Policy and regulatory 3. Training 4. Support and supply chain 5. Quality assurance 6. Connectivity and IT 7. Impact measurement | Follows the key elements of launching and scaling up a specific TB diagnostic and highlights gaps along the pathway that have hindered progress. | Highlights that while data has improved, more data are needed to answer critical questions (e.g., patient and public health impact, potential cost savings, etc). |
| **USAID** *(2015)* [8] | Global health innovations (including diagnostics) | 1. Identify need and design 2. Begin research and development 3. Plan for introduction 4. Introduce and scale | Aims to identify best practices for scaling innovations in global health, listing the key stages and the priority activities through case studies and lessons learned. |  |

**References**

1. World Economic Forum. Diagnostics for better health: considerations for global implementation. Cologny/Geneva: World Economic Forum. 2021. Available from: <https://www.weforum.org/reports/diagnostics-for-better-health-considerations-for-global-implementation/>.

2. Mugambi ML, Peter T, S FM, Giachetti C. How to implement new diagnostic products in low-resource settings: an end-to-end framework. BMJ Glob Health. 2018;3(6):e000914.

3. Derda R, Gitaka J, Klapperich CM, Mace CR, Kumar AA, Lieberman M, et al. Enabling the Development and Deployment of Next Generation Point-of-Care Diagnostics. PLoS Negl Trop Dis. 2015;9(5):e0003676.

4. Engel N, Wachter K, Pai M, Gallarda J, Boehme C, Celentano I, et al. Addressing the challenges of diagnostics demand and supply: insights from an online global health discussion platform. BMJ Glob Health. 2016;1(4):e000132.

5. Research Institute of the McGill University Health Centre. TB diagnostics critical pathway [Internet]. 2017. Available from: <http://www.tbdxpathway.org/>.

6. Stop TB Partnership, World Health Organization. Pathways to better diagnostics for tuberculosis: a blueprint for the development of TB diagnostics by the new diagnostics working group of the Stop TB Partnership. Geneva: World Health Organization. 2009. Available from: <https://apps.who.int/iris/handle/10665/44230>.

7. Albert H, Nathavitharana RR, Isaacs C, Pai M, Denkinger CM, Boehme CC. Development, roll-out and impact of Xpert MTB/RIF for tuberculosis: what lessons have we learnt and how can we do better? Eur Respir J. 2016;48(2):516-25.

8. U.S. Agency for Interntational Development (USAID). Idea to impact: A guide to introduction and scale of global health innovations Washington, DC: USAID. 2015. Available from: <https://www.usaid.gov/document/idea-impact-guide-introduction-and-scale-global-health-innovation>.
