# Supplementary material for: Defining a malaria diagnostic pathway from innovation to adoption: Stakeholder perspectives on data and evidence gaps
Source: PLOS Glob Public Health. 2024 May 16;4(5):e0002957. doi: 10.1371/journal.pgph.0002957 (PMC11098419; doi:10.1371/journal.pgph.0002957)
Supplement: S3 Text — (DOCX) [file pgph.0002957.s003.docx]

**S2 Table. Data and ranking for Fig 1**

| Stage | N (n)** | Attribute | Strongly disagree | Somewhat disagree | Neither agree nor disagree | Somewhat agree | Strongly agree | Disagree (at least somewhat) | Disagree rank* | Agree  (at least somewhat) | Agree rank* |
| --- | --- | --- | --- | --- | --- | --- | --- | --- | --- | --- | --- |
| Needs assessment | 119  (43) | Clear requirements | 6% | 19% | 6% | 49% | 20% | 25% | 2 | 69% | 3 |
|  |  | Meets user needs | 3% | 22% | 14% | 50% | 11% | 25% | 2 | 61% | 4 |
|  |  | Accurate & reliable | 2% | 25% | 22% | 42% | 9% | 27% | 2 | 51% | 5 |
|  |  | Publicly/freely available | 7% | 24% | 19% | 44% | 6% | 31% | 3 | 50% | =2 |
| Feasibility, development, & validation | 121  (55) | Clear requirements | 6% | 13% | 5% | 56% | 20% | 19% | 4 | 76% | 1 |
|  |  | Meets user needs | 4% | 13% | 10% | 54% | 19% | 17% | 5 | 73% | 1 |
|  |  | Accurate & reliable | 5% | 13% | 13% | 47% | 21% | 18% | 4 | 69% | 2 |
|  |  | Publicly/freely available | 13% | 13% | 15% | 43% | 16% | 26% | 4 | 59% | 1 |
| Approvals & manufacturing | 19  (10) | Clear requirements | 16% | 21% | 5% | 37% | 21% | 37% | 1 | 58% | 6 |
|  |  | Meets user needs | 16% | 21% | 16% | 16% | 32% | 37% | 1 | 47% | 6 |
|  |  | Accurate & reliable | 21% | 21% | 11% | 26% | 21% | 42% | 1 | 47% | 6 |
|  |  | Publicly/freely available | 32% | 16% | 11% | 32% | 11% | 47% | 1 | 42% | 6 |
| Preparation for launch | 26  (12) | Clear requirements | 8% | 12% | 15% | 54% | 12% | 19% | 3 | 65% | 4 |
|  |  | Meets user needs | 8% | 12% | 15% | 42% | 23% | 19% | 4 | 65% | 3 |
|  |  | Accurate & reliable | 8% | 8% | 15% | 58% | 12% | 15% | 5 | 69% | 1 |
|  |  | Publicly/freely available | 15% | 15% | 19% | 50% | 0% | 31% | 2 | 50% | =2 |
| Adoption & scale-up | 82  (34) | Clear requirements | 4% | 7% | 16% | 61% | 12% | 11% | 6 | 73% | 2 |
|  |  | Meets user needs | 4% | 10% | 19% | 59% | 9% | 14% | 6 | 68% | 2 |
|  |  | Accurate & reliable | 4% | 11% | 22% | 56% | 7% | 15% | 6 | 63% | 3 |
|  |  | Publicly/freely available | 5% | 16% | 32% | 41% | 6% | 21% | 6 | 48% | 5 |
| Surveillance & impact measurement | 83  (56) | Clear requirements | 2% | 16% | 20% | 43% | 18% | 18% | 5 | 61% | 5 |
|  |  | Meets user needs | 4% | 16% | 23% | 45% | 13% | 19% | 3 | 58% | 5 |
|  |  | Accurate & reliable | 4% | 16% | 21% | 45% | 15% | 20% | 3 | 60% | 4 |
|  |  | Publicly/freely available | 6% | 19% | 25% | 40% | 10% | 25% | 5 | 49% | 4 |

*Ranked from 1-6 where 1=Highest level of disagreement/agreement and 6=Lowest level of disagreement/agreement

***N*=total number of activities respondents were involved in contributing to the results and *n*=number of individual respondents
